# Fly Exterminator

**Published:** 1876-05

**Authors:** 


### FLY EXTERMINATOR.

THE best is cleanly housekeeping and darkened dining-rooms. If the common "Plantain" or *Fleawort* is dipped in milk and hung up in a room, it will banish the flies.

THE CHINESE LINDEN is an early, and most beautiful shade-tree. Worms eat off its first crop of leaves; but they put out again—and during the summer and fall, the ground or pavement under it is covered with dead house-flies, although swept away every day; so that, between darkened rooms, cleanly housekeeping, and the Chinese linden before our door, we are very little troubled with flies.

Unless our subscribers intend to enrich LANDON of Hudson street, we would advise them, as the most effectual means of preserving their carpets from moths, to dampen a cloth, lay it over the carpet, especially at its edges and most unused spots, and run over it with a hot smoothing-iron. Do it at once, and repeat the operation two or three times during the warm weather. The hot steam kills the eggs.

